# Supplementary material for: Effect of a Rumen-Protected Microencapsulated Supplement from Linseed Oil on the Growth Performance, Meat Quality, and Fatty Acid Composition in Korean Native Steers
Source: Animals (Basel). 2021 Apr 27;11(5):1253. doi: 10.3390/ani11051253 (PMC8145495; doi:10.3390/ani11051253)
Supplement: Supplementary file 1 [file animals-11-01253-s001.zip › animals-1185581-supplementary.pdf]

Scheme 1. Hematological analyses of blood in steers supplemented with concentrate containing 3% rumen-protected microencapsulated supplement (MO).

Items	CON	3% MO [1]	*p*-Value
	Lymphocyte (2.5–7.5 K/uL)		
Day 0	7.87	9.46	0.463
Day180	4.93	5.55	0.480
	Monocyte (0–0.84 K/uL)		
Day 0	0.6	0.74	0.444
Day180	0.32	0.11	0.267
	Granulocyte (0.6–6.7 K/uL)		
Day 0	2.46	2.19	0.729
Day180	3.33	3.60	0.656
	White blood cell (4–12 K/uL)		
Day 0	10.93	12.39	0.462
Day180	8.58	9.26	0.613
	Red blood cell (5–10 M/uL)		
Day 0	9.08	8.12	0.249
Day180	8.72	8.27	0.556
	Hemoglobin (8–15 g/dL)		
Day 0	15.45	15.15	0.601
Day180	13.78	13.8	0.982
	Hematocrit (24–46%)		
Day 0	38.15	37.22	0.497
Day180	37.75	38.79	0.735
	Mean corpuscular volume (40–60 fL)		
Day 0	44	45.33	0.419
Day180	45	47.5	0.276
	Mean corpuscular hemoglobin (11–17 pg)		
Day 0	16.58	17.73	0.354
Day180	15.17	16.44	0.422
	Mean corpuscular hemoglobin concentration (30–36 g/dL)		
Day 0	40.48	40.60	0.796
Day180	35.8	35.5	0.377
	Platelet (100–800 K/ul)		
Day 0	333	292.2	0.166
Day180	264.5	267	0.894

Values are express as means (n = 4). [1] 3% MO, 3% MO supplementation to concentrate.
